# Radiofrequency ablation of premature ventricular contractions guided by robotic magnetic navigation combined with pattern matching filter

**DOI:** 10.1002/clc.24010

**Published:** 2023-03-23

**Authors:** Xiao‐yu Liu, Jie Zheng, Ku‐lin Li, Shi‐peng Dang, Xiao‐yan Li, Xiao‐xi Zhao, Chao Wang, Zhi‐ming Yu, Ru‐xing Wang

**Affiliations:** ^1^ Department of Cardiology Wuxi People's Hospital affiliated to Nanjing Medical University Wuxi China

**Keywords:** ablation, pattern matching filter, premature ventricular contractions, robotic magnetic navigation

## Abstract

**Background:**

This study's intent is to evaluate the usefulness of pattern matching filter (PMF) function combined with robotic magnetic navigation (RMN) in guiding the ablation of premature ventricular contractions (PVCs).

**Hypothesis:**

Assume that PMF can improve the outcomes of PVCs ablation using RMN.

**Methods:**

A retrospective analysis was completed consisting of 118 consecutive patients with PVCs who underwent radiofrequency ablation guided by RMN. According to the application of PMF, patients were divided into two groups: 20 patients underwent ablation without PMF (group A), and another 98 patients received ablation incorporating PMF (group B).

**Results:**

Compared with group A, the procedure time (135.0 ± 28.3 min vs. 106.3 ± 37.9 min, *p* = 0.02) in group B was significantly decreased, while the X‐ray exposure time (6.0 ± 2.6 min vs. 6.5 ± 3.6 min, *p* = 0.705) and dose (3.2 ± 2.4 gycm^2^ vs. 3.9 ± 2.7 gycm^2^，*p* = 0.208) had no significant difference. Group B had a more than twofold number of points acquired (66.9 ± 23.0 vs. 143.9 ± 68.3, *p* < 0.001) and required a shorter radiofrequency ablation time (13.2 ± 3.5 min vs. 8.1 ± 2.9 min, *p* < 0.001). There were no serious complications in either group. The acute success rate was similar [90.0% (18/20) vs. 87.8% (86/98), *p* = 1.000] in two groups, and the success rate was also similar in the long‐term follow‐up [83.3% (15/18) vs. 87.2% (75/86), *p* = 0.776].

**Conclusions:**

The ablation of PVCs guided by RMN is safe and effective. Combined with the functional capability of PMF, both procedure time and radiofrequency ablation time were significantly decreased.

## Graphical abstract

Robotic magnetic navigation (RMN) is an acknowledged tool for premature ventricular contractions (PVCs) ablation. Pattern matching filter (PMF) is a new functional software which enables a correlation to be made between the real time PVCs and the predefined template morphology. PVCs ablation using RMN combined with the function of PMF is safe and effective, and both total procedure time and ablation time are significantly reduced.

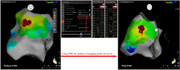

## 1 INTRODUCTION

Radiofrequency ablation therapy guided by robotic magnetic navigation (RMN) is a well‐studied and acknowledged treatment for premature ventricular contractions (PVCs) and other complex arrhythmias with high efficacy, fewer complications and less X‐ray exposure versus traditional manual catheter ablation, and a short technology learning curve.^1^, ^2^, ^3^, ^4^, ^5^, ^6^, ^7^ Since 2013, our center has been employing RMN combined with three‐dimensional electroanatomic mapping system (CARTO, Biosense Webster Inc) in ablation therapy across a wide range of complex arrhythmias including PVCs and ventricular tachycardia. During our center's early experience, the mapping aspect of PVCs procedures presented challenges. With the innovation of CARTO technology, new functional software called pattern matching filter (PMF, Biosense Webster Inc) has been offered which enables a correlation to be made between the real time PVCs and the predefined template morphology. Limited studies have reported that PMF can improve the outcomes in manually controlled catheter ablation.^8^ The object of this study was to evaluate the usefulness of PMF in combination with RMN for PVCs ablation.

## 2 MATERIALS AND METHODS

### 2.1 Study population

Between May 2017 and May 2022, in Wuxi People's Hospital affiliated to Nanjing Medical University, a total of 118 patients with symptomatic and medically refractory PVCs underwent first time ablation therapy using RMN and were included in this retrospective study. All procedures were performed using RMN in our center due to the system's superior safety and efficacy (Before May 2017, our center had completed ablation of complex arrhythmia guiding by RMN for more than 150 cases.).^9^, ^10^ Routine blood, coagulation index, kidney and liver function, as well as echocardiography and electrocardiogram were performed for all patients before their procedures. Patients with hypertrophic cardiomyopathy, coronary heart disease with previous history of myocardial infarction or interventional therapy, dilated cardiomyopathy, history of cardiac surgery, left ventricular ejection fraction (LVEF) <40%, NYHA class III‐IV, serious renal dysfunction and serious hepatic dysfunction were excluded in this study. The clinical baseline characteristics of patients are listed in Table 1. Informed consent was obtained for all patients before the procedure. The investigations were in accordance with the Declaration of Helsinki and got the ethics approval from the Ethics Committee of Wuxi People's Hospital. Based on the application of PMF function, patients were divided into two groups. Ablation was performed in 20 patients without PMF (Group A), and another 98 patients using PMF (Group B).

**Table 1:** Baseline characteristics of the enrolled patients.

	Group A (*n* = 20)	Group B (*n* = 98)	Total (*n* = 118)	*p* Value
Gender (female/male)	13/7	56/42	69/49	0.516
Age (years)	47.5 ± 20.0	54.5 ± 15.9	53.3 ± 16.7	0.087
Height (cm)	168.8 ± 9.1	166.8 ± 8.9	167.2 ± 8.9	0.338
Weight (kg)	67.4 ± 11.7	66.7 ± 10.6	66.9 ± 10.8	0.805
BMI (kg/m^2^)	23.7 ± 4.1	24.0 ± 3.5	23.9 ± 3.6	0.756
PVCs burden (thousand/24 h)	25.8 ± 13.4	26.4 ± 11.2	26.3 ± 11.6	0.744
Course of PVCs (month)	30.3 ± 32.8	42.7 ± 68.4	40.6 ± 63.8	0.965
Basic diseases	5	45	50	0.084
Hypertension	4	37	41	0.207
Diabetes	0	11	11	0.250
Coronary heart disease	2	11	13	1.000
LVEF (%)	63.3 ± 2.8	62.3 ± 3.5	62.5 ± 3.4	0.321
Antiarrhythmic drugs	1.2 ± 0.4	1.1 ± 0.3	1.1 ± 0.4	0.321

*Note*: *p* Values listed were calculated between Group A and B.

Abbreviations: BMI, body mass index; LVEF, left ventricular ejection fraction; PVCs, premature ventricular contractions.

### 2.2 Mapping and ablation

After five half‐lives withdrawal of antiarrhythmic drugs, procedures were performed with patients in a fasting and conscious state. Intracardiac electrograms and 12‐lead ECGs were recorded by a multichannel digital mapping system. PVCs were induced by intravenous isoproterenol infusion if they failed to occur spontaneously. l. In all patients, via the femoral vein, a 6F decapolar coronary sinus catheter (Inquiry, St. Jude Medical, Inc) was placed in the coronary sinus and a 6F quadrupole catheter (Inquiry, St. Jude Medical, Inc) was placed at the right ventricular apex.

For right‐sided procedures, a RMN catheter (Navistar Thermocool RMT, Biosense Webster, Inc) was advanced into the right ventricle (RV). For left‐sided procedures, a transseptal puncture was the preferred access choice, but utilizing a retrograde approach was also permitted when deemed appropriate. Heparin was administered intravenously to maintain an activated clotting time between 250 and 350 s.

The RMN Niobe® ES system (Stereotaxis Inc) was used in conjunction with CARTO to perform mapping.^11^ The model of the RV/left ventricle (LV) was constructed under structure mapping model in both groups, and then patients underwent electro‐anatomical mapping point by point.

**Figure 1:**
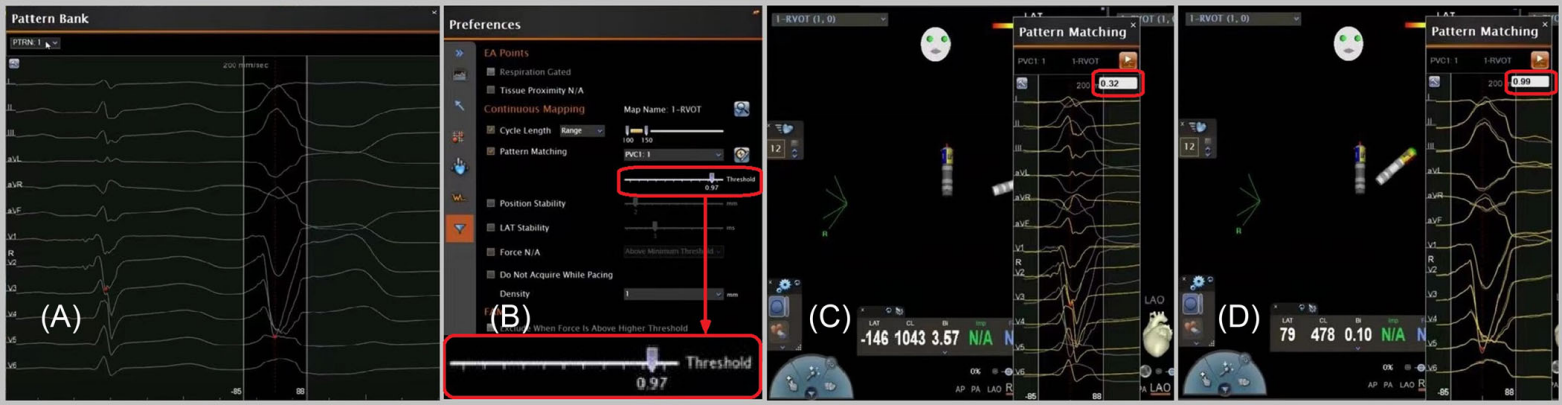
Pattern matching filter (PMF) setup workflow. (A) Selection of the premature ventricular contractions (PVCs) as the template morphology to be mapped in the pattern bank. The window of interest was adjusted to include only the QRS complex. (B) A 97% minimum threshold was selected and during continuous mapping, each PVCs and its respective correlation were visualized. (C) If the QRS complex presented a correlation ≥97%, activation points were collected. (D) If the QRS complex presented a correlation.

In the PMF mapping process, the first step was to collect 12.5 s 12‐lead ECG information in the pattern bank of the Carto system, including at least one clinical PVCs (Figure 1A). Second, the target PVCs was selected in the pattern bank window and included only one QRS complex of the target PVCs in the range of the PMF interest window (Figure 1A). Third, the PMF function was set and enabled in the preferences user interface, and the correlation percentage of 97% selected (Figure 1B). When the morphological similarity between the real time PVCs and the template PVCs is at or above 95%, the system automatically takes a point for activation mapping (Figure 1C,D). After completing the above steps, the RMN catheter was navigated around the ventricular 3D map and the PMF function automatically obtained points during activation mapping. Subsequently, the earliest activation point gradually appeared in the 3D space. Finally, when required pace mapping was used to confirm the earliest activation point of the PVCs. In the non‐PMF group A, instead of leveraging software the mapping process required the traditional manual method of taking points when the target PVCs presented.

Ablation points were delivered according to the following criteria and all criteria were met at the same time: local activation at least 20 ms before QRS, greater than 95% of QRS matches during pacing mapping, and unipolar ECG assumed QS morphology. The radiofrequency (RF) power setting ranged from 20 to 40 W, depending on the region being targeted. The temperature control setting was set with an upper limit of 43°C, and the flow setting of normal saline during ablation was 17–20 mL/min. If PVCs reduction or elimination was observed within 10 s after ablation energy delivery, we continued to ablate for 90–120 s, otherwise terminated ablation, and mapped to find the new ablation site.^12^ After the first ablation, isoproterenol was injected intravenously, and the patient observed for 30 min. If there was no PVCs recurrence, the procedure was defined as an acute success. In the event of PVCs recurrence, further diagnosis and ablation continued. If all identified ablation points were ablated and this did not terminate the PVCs, the procedure was defined as unsuccessful.

Procedure‐related parameters were recorded, including X‐ray exposure time, X‐ray dose, procedure time, ablation time, and complications. Procedure time was defined as the time from the first femoral vein puncture to the sheath removal.

### 2.3 Follow‐up

ECG monitoring was performed from the beginning of procedure to hospital discharge. Following the procedure, patients underwent follow‐up every 3 months in an outpatient clinic. 24 h Holter ECG recording was examined routinely within the 3 months intervals. Patients were directed to return to the hospital for further consultation if they experienced any symptomatic palpitations. Recurrence was defined as asymptomatic and/or symptomatic PVCs confirmed by Holter ECG recordings. In the event the same clinical PVCs recurred, the procedure was not classified as a long‐term success.

### 2.4 Complications

Complications were classified into two categories: major and minor. Major complications included stroke, acute myocardial infarction, high grade atrioventricular block, pericardial effusion/cardiac tamponade, and major bleeding. Minor complications included inguinal hematoma and puncture point infection.

### 2.5 Statistical analysis

SPSS v22.0 statistical software was used for analysis. Continuous variables with normal distribution were expressed as mean ± standard. Categorical variables were expressed as a percentage. The Student's *t*‐test was performed for comparison of continuous variables with normal distribution. The nonparametric test (Mann–Whitney) was performed for continuous variables with non‐normal distribution. Categorical variables were compared with the Fisher's exact test or *χ* ^2^. Kaplan–Meier survival function was performed for analyzing the event‐free survival rate, and differences of the groups were assessed by the Log‐Rank test. *p* < 0.05 was defined as statistically significant difference.

## 3 RESULTS

### 3.1 Baseline of patients

The clinical characteristics of the patients are shown in Table 1. No significant difference was found in the baseline characteristics between Group A and Group B.

### 3.2 Characteristics of mapping and ablation

Characteristics of mapping and ablation are shown in Table 2 and Supporting Information: Figure S1. Compared with Group A, the procedure time (135.0 ± 28.3 min vs. 106.3 ± 37.9 min, *p* = 0.02) in Group B was significantly decreased. However, the X‐ray exposure time (6.0 ± 2.6 min vs. 6.5 ± 3.6 min, *p* = 0.705) and dose (3.2 ± 2.4 gycm^2^ vs. 3.9 ± 2.7 gycm^2^，*p* = 0.208) had no significant difference. Group B had a more than twofold increase in the number of points acquired (66.9 ± 23.0 vs. 143.9 ± 68.3, *p* < 0.001) and required a shorter radiofrequency ablation time (13.2 ± 3.5 min vs. 8.1 ± 2.9 min, *p* < 0.001). There were no serious complications in either group.

**Table 2:** Procedural parameters.

	Group A (*n* = 20)	Group B (*n* = 98)	Total (*n* = 118)	*p* Value
Procedure duration (min)	135.0 ± 28.3	106.3 ± 37.9	111.2 ± 37.9	0.02
ablation time (min)	13.2 ± 3.5	8.1 ± 2.9	8.9 ± 3.6	<0.001
Total X‐ray time (min)	6.0 ± 2.6	6.5 ± 3.6	6.5 ± 3.4	0.705
Total X‐ray dose (gycm^2^)	3.2 ± 2.4	3.9 ± 2.7	3.7 ± 2.7	0.208
Points in activation mapping model	66.9 ± 23.0	143.9 ± 68.3	130.8 ± 69.2	<0.001
Complications (minor)	0	0	0	—
Complications (major)	0	0	0	—
Ablation point advance QRS (ms)	30.0 ± 4.9	30.8 ± 5.1	30.7 ± 5.1	0.503

*Note*: *p* Values listed were calculated between Group A and B.

### 3.3 Distribution of PVCs

The distribution of PVCs origin points is listed in Supporting Information: Table S1. A considerable percentage of PVCs originate from the outflow tract (OT) of either the RV or the LV, and this study yielded similar results.^13^ The proportion of PVCs originating from the RVOT alone accounted for more than half at 57.6% (68/118). Therefore, in our analysis PVCs were generally divided into RVOT origin and non‐RVOT origin for interpretation of results.

### 3.4 Procedural outcomes

**Figure 2:**
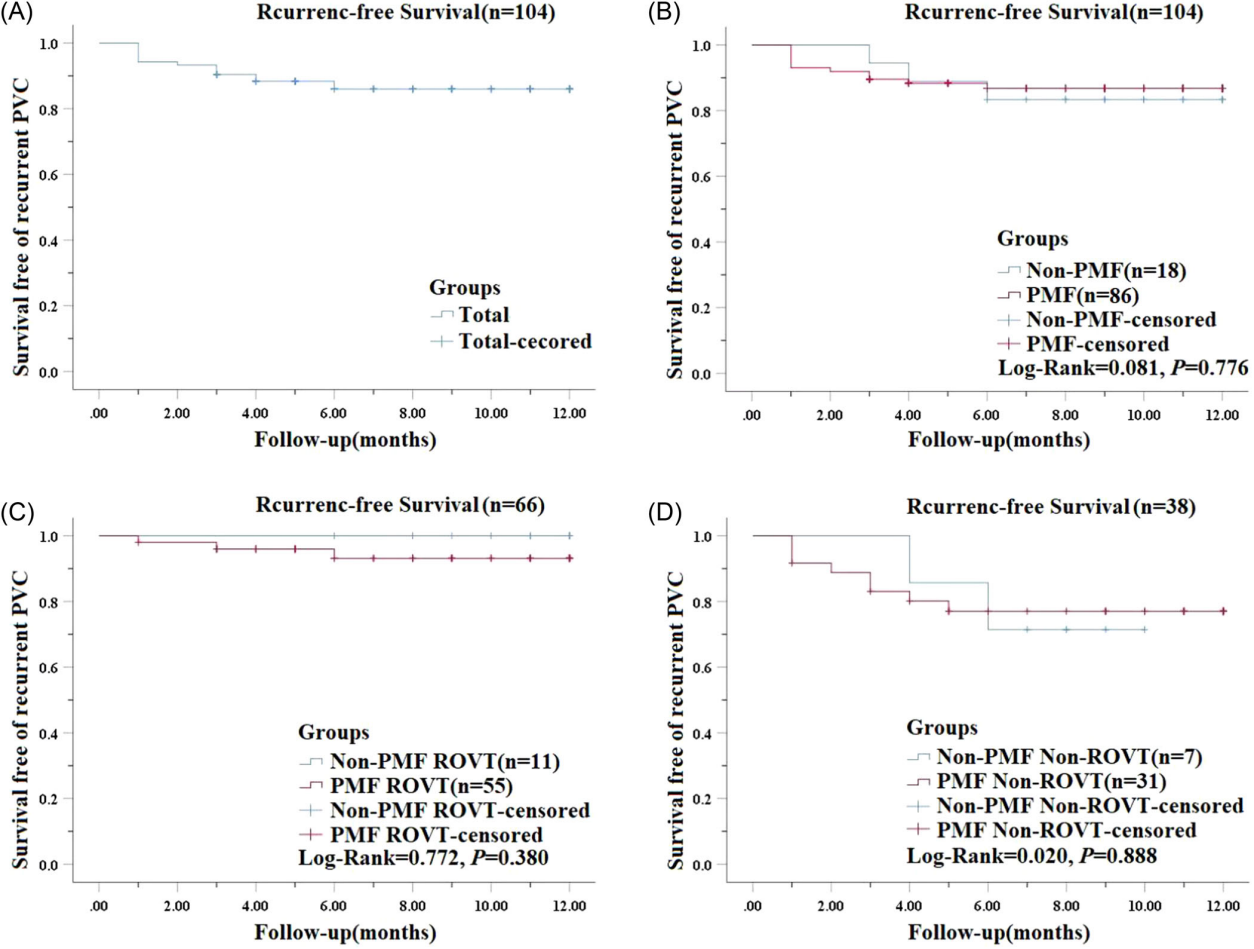
Kaplan–Meier curves of recurrence‐free survival after primary successful ablation. (A) Kaplan–Meier curves demonstrate recurrence‐free survival for patients that underwent premature ventricular contractions (PVCs) ablation. (B) Kaplan–Meier curves demonstrate recurrence‐free survival for patients that underwent PVCs ablation with and without pattern matching filter (PMF) (*p* = 0.776). (C) Kaplan–Meier curves demonstrate recurrence‐free survival for patients that underwent right ventricular outflow tract (RVOT) PVCs ablation with and without PMF (*p* = 0.772). (D) Kaplan–Meier curves demonstrate recurrence‐free survival for patients that underwent non‐RVOT PVCs ablation with and without PMF (*p* = 0.380).

Procedural outcome parameters of acute success and recurrence during follow‐up are listed in Table 3 and Figure 2. The acute success rate [90.0% (18/20) vs. 87.8% (86/98), *p* = 1.000] and long‐term success rate [83.3% (15/18) vs. 87.2% (75/86), *p* = 0.776] were similar in both groups. The results demonstrated that there was no significant difference in the acute success rate [91.7% (11/12) vs. 98.2% (55/56), *p* = 0.782] and long‐term success rate [100% (11/11) vs. 94.5% (52/55), *p* = 0.380] of PVCs in the ROVT between the two groups. There was also no significant difference in the acute success rate [87.5% (7/8) vs. 73.8% (31/42), *p* = 0.704] and long‐term success rate [57.1% (4/7) vs. 74.2% (23/31), *p* = 0.888] of PVCs in the non‐ROVT between the two groups. The distribution of ventricular original points in cases with unsuccessful acute ablation and recurrence cases during follow‐up is listed in Supporting Information: Table S2. It is obvious that the acute success rate of PVCs from ROVT is higher than that of non‐ROVT PVCs [97.1% (66/68) vs. 76.0% (38/50), *p* < 0.001], and the long‐term success rate is also significantly higher than that of non‐ROVT PVCs [95.5% (63/66) vs. 71.1% (27/38), *p* < 0.001].

**Table 3:** Outcomes of ablation.

	Group A (*n* = 20)	Group B (*n* = 98)	Total (*n* = 118)	*p* Value
Follow‐up (month)	8.9 ± 2.8	7.9 ± 3.6	8.1 ± 3.5	0.228
Actue‐term success (%)	90.0 (18/20)	87.8 (86/98)	88.1 (104/118)	1.000
RVOT (%)	91.7 (11/12)	98.2 (55/56)	97.1 (66/68)	0.782
Non‐RVOT (%)	87.5 (7/8)	73.8 (31/42)	76.0 (38/50)	0.704
Long‐term success (%)	83.3 (15/18)	87.2 (75/86)	86.5 (90/104)	0.776
RVOT (%)	100 (11/11)	94.5 (52/55)	95.5 (63/66)	0.380
Non‐RVOT (%)	57.1 (4/7)	74.2 (23/31)	71.1 (27/38)	0.888

*Note*: *p* Values listed were calculated between group A and B.

Abbreviation: RVOT, right ventricular outflow tract.

## 4 DISCUSSION

To the best of our knowledge, this is the first study focusing on PVCs ablation using the RMN system in combination with PMF function. The main findings of this study are three‐fold. First, with the employment of PMF function, more effective mapping points can be obtained resulting in more detailed and accurate activation mapping images. Second, combined with PMF the procedural time and radiofrequency ablation time of PVCs ablation using RMN were reduced. Third, no changes were found in either acute or long‐term success rates.

Radiofrequency ablation guided by RMN is a safe and effective way to treat PVCs.^2^, ^14^, ^15^, ^16^, ^17^ The omnidirectional flexible magnetic catheter is not restricted by fixed curves and can reach most any challenging anatomical position.^3^ Additionally, once the operator reaches the desired location the soft and magnetic properties of the catheter allow the tip to stay in the same location throughout the cardiac cycle. This unique tip‐tissue dynamic provides greater catheter stability and possibly negates the influence of an input that is difficult to measure: variation in manual dexterity between operators and the associated impact on safety and efficacy.^18^, ^19^ Several studies have shown similar success rates for PVCs ablation under the guidance of RMN when compared to manual ablation.^16^, ^20^ The key advantage of RMN ablation is consistent reduction in X‐ray exposure for physicians and patients, and no serious complications were reported.^16^, ^21^, ^22^ Our center began to use RMN for ablation treatment of complex arrhythmias form 2013, including PVCs ablation. However, in the actual work process, we found that PVCs ablation takes a lot of time in the mapping process.

Due to the single tip of the RMN catheter, it can only carry out point by point activation mapping. Therefore, mapping points selected is time‐consuming. When there is no PMF support, it requires several steps of operation. First, move the catheter tip to the position of interest. Second, after the tip of catheter stays at the interest point, wait for the PVCs appearance. Third, take a point when the PVCs appearance occurs. These complex operations lead to prolonged procedure time, and the number of taken points is limited. When the PMF function is turned on, the software automatically takes points according to the parameters in the process of moving the catheter, which saves the procedure time and increases the number of taken points per unit time.

During the activation mapping, only points with a correlation of more than 97% were automatically added to the model by setting a high correlation threshold with the PVCs morphology to be mapped. This high correlation threshold alleviates the trigger of mechanical PVCs caused by the movement of the catheter, which means each point during the activation mapping is highly reliable.

According to the principle of activation mapping, higher density of effective points obtained on the unit area of model is conducive to ablation treatment. When the PMF function is applied, the number of points obtained on the unit area of model increased significantly, which leads to an increase in the accuracy of identification of the earliest activation point (Supporting Information: Figure S1). Then, the accuracy of radiofrequency ablation energy delivery was also increased. Therefore, the duration of ablation energy delivery in PMF using group reduced.

In terms of outcomes, no statistically significant results were found. Compared with the success rate of manual ablation reported in previous studies (ranging from 50% to >90%), there is no significant difference in the success rate of PVCs ablation using RMN in our center, which is consistent with our expectations.^15^, ^16^ However, our study shows that the application of PMF function failed to improve the acute success rate and failed to reduce the recurrence, which is inconsistent with our expectations. After analyzing the data, we found that we conducted detailed mapping for all patients as much as possible without PMF. Although the mapping was time‐consuming and slightly less accurate than that in the PMF group, the increased energy delivery time made up for the lack of accuracy of mapping. According to previous studies, success rates with PVCs ablation can vary according to PVCs sites of origin, electrophysiological characteristics, ablation techniques, radiofrequency energy delivery and experience of operator.^7^, ^13^ So we suggest that PMF can only help us obtain mapping points quickly, shorten the procedure time.

Other 3D mapping systems also have similar functions, such as the automark function of EnSite system. However, there is no relevant research on the clinical application of automark function. So, it is difficult to make comparation. The report of animal experiment indicates that the automatic mapping function is significantly faster than manual point‐by‐point 3D mapping and results in shorter mapping time and higher point density.^23^ Although the automatic mapping function cannot increase the success rate of surgery, the reduction of operation time can also bring benefits to patients, such as reducing the pain and psychological burden, and potentially reducing the possibility of complications. For the operator, reducing the operation time means that more patients can be treated in unit time.

In terms of X‐ray application, the results of our center showed that the X‐ray exposure of RMN ablation was limited. There was no significant difference in X‐ray exposure between the two groups because the application of X‐ray was not involved in the mapping stage.

## 5 LIMITATIONS

Limitations for this study are acknowledged as follows. First, RMN ablation of PVCs combined with the PMF should be further evaluated in randomized controlled trials and compared to manual ablation. Second, the number of patients in this study was relatively small, and therefore the comparison is inherently underpowered. Third, this study is limited by the inherent nature of a retrospective study and the results need to be confirmed by prospective studies. Fourth, it is unknow about the PVC burden during procedure in both groups. Therefore, the comparison of the number of acquired points in unit time may exist statistical bias.

## 6 CONCLUSION

In conclusion, PVCs ablation using RMN combined with the function of PMF is safe and effective. Utilizing the PMF function, both total procedure time and ablation time are significantly reduced. However, RMN itself is still the technology driving safety and efficacy of the ablation procedure.

## CONFLICT OF INTEREST STATEMENT

The authors declare no conflict of interest.

## Supporting information

Supplementary information.

Supplementary information.

Supplementary information.

## Data Availability

The figure and table data used to support the findings of this study are included within the article.
