# Supplementary figures and images for: Radiofrequency ablation of premature ventricular contractions guided by robotic magnetic navigation combined with pattern matching filter
Source: Clin Cardiol. 2023 Mar 23;46(5):567–73. doi: 10.1002/clc.24010 (PMC10189081; doi:10.1002/clc.24010)

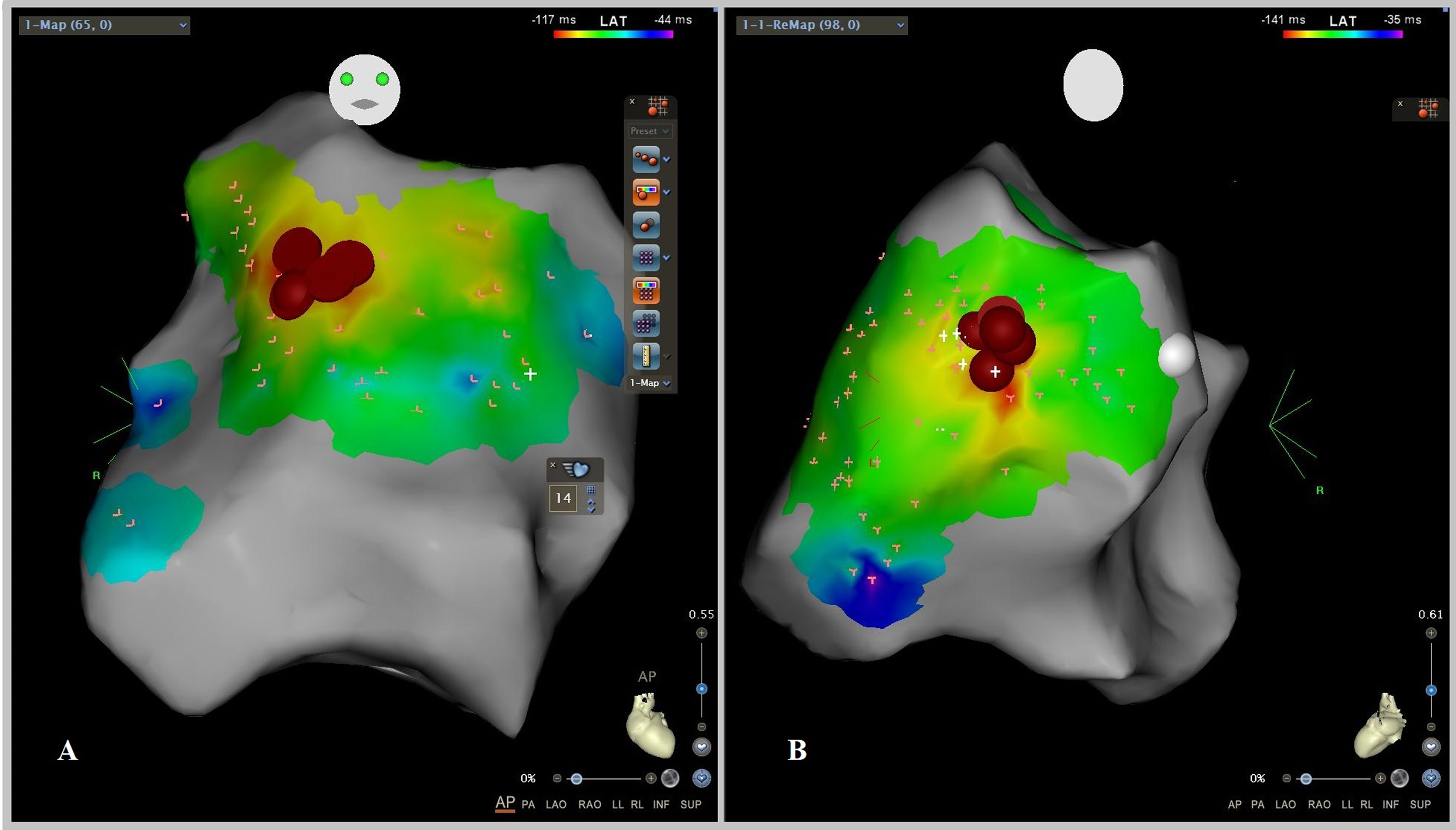

Supplement: Supplementary file 1 — Supplementary information. [file CLC-46-567-s003.tif]
