# Supplementary material for: Radiofrequency ablation of premature ventricular contractions guided by robotic magnetic navigation combined with pattern matching filter
Source: Clin Cardiol. 2023 Mar 23;46(5):567–73. doi: 10.1002/clc.24010 (PMC10189081; doi:10.1002/clc.24010)
Supplement: Supplementary file 2 — Supplementary information. [file CLC-46-567-s001.doc]

**Table S1 Location of premature ventricular contractions**

|  | Group A (n=20) | Group B (n=98) | Total (n=118) | *P* value |
| --- | --- | --- | --- | --- |
| RV |  |  |  |  |
| RVOT | 12 | 56 | 68 | 0.814 |
| Tricuspid annulus | 2 | 13 | 15 | 0.975 |
| Septal wall | -- | 1 | 1 | 1.000 |
| LV |  |  |  |  |
| LVOT including coronary cusps | 3 | 4 | 7 | 0.172 |
| Mitral annulus | -- | 3 | 3 | 1.000 |
| Aortomitral continuity | -- | 3 | 3 | 1.000 |
| Papillary | -- | 2 | 2 | 1.000 |
| Left anterior fascicular | -- | 2 | 2 | 1.000 |
| Left posterior fascicular | -- | 2 | 2 | 1.000 |
| Septal wall | 1 | 3 | 4 | 0.529 |
| Para-Hisian | 2 | 5 | 7 | 0.745 |
| Coronary sinus | -- | 4 | 4 | 1.000 |

*P* values listed were calculated between group A and B. RV: right ventricular, LV: left ventricular, OT: outflow tract
