# Supplementary material for: Radiofrequency ablation of premature ventricular contractions guided by robotic magnetic navigation combined with pattern matching filter
Source: Clin Cardiol. 2023 Mar 23;46(5):567–73. doi: 10.1002/clc.24010 (PMC10189081; doi:10.1002/clc.24010)
Supplement: Supplementary file 3 — Supplementary information. [file CLC-46-567-s002.doc]

**Table S2 Actual unsuccess and recurrence cases**

|  | Actual unsuccess cases | | Recurrence of actual success cases | |
| --- | --- | --- | --- | --- |
|  | Group A (n=20) | Group B (n=98) | Group A (n=18) | Group B (n=86) |
| RV |  |  |  |  |
| RVOT | 1 | 1 | -- | 3 |
| Tricuspid annulus | 1 | 2 | -- | 4 |
| Septal wall | -- | -- | -- | 1 |
| LV |  |  |  | -- |
| LVOT including coronary cusps | -- | 2 | 1 | -- |
| Mitral annulus | -- | 1 | -- | -- |
| Aortomitral continuity | -- | -- | -- | -- |
| Papillary | -- | 2 | -- | -- |
| Left anterior fascicular | -- | -- | -- | 1 |
| Left posterior fascicular | -- | -- | -- | -- |
| Septal wall | -- | -- | -- | -- |
| Para-Hisian | -- | 1 | 2 | 1 |
| Inside coronary sinus | -- | 3 | -- | 1 |

RV: right ventricular, LV: left ventricular, OT: outflow tract
